# 

**Published:** 1896-07

**Authors:** 


PUBLISHED BY THE FRANKLIN PRINTING AND PUBLISHING CO., ATLANTA, GA.
Entered at the Post-Office at Atlanta, Ga., as second-class matter.

*We positively cannot send premium to any subscriber who does not pay a year in advance.*

NOTICE.—Our offer of a thermometer to *all* cash-in-advance subscribers is withdrawn. *New* subscribers, paying full year in advance, may still get the thermometer. Book offer still open to all who pay a year in advance. "Stories of a Country Doctor."

